# Condom use peer norms and self-efficacy as mediators between community engagement and condom use among Chinese men who have sex with men

**DOI:** 10.1186/s12889-017-4662-4

**Published:** 2017-08-07

**Authors:** Haochu Li, Li Xue, Joseph D. Tucker, Chongyi Wei, Maya Durvasula, Wenqi Hu, Dianming Kang, Meizhen Liao, Weiming Tang, Wei Ma

**Affiliations:** 1 Department of Epidemiology, School of Public Health, Shandong University, 44 West Wenhua Road Shandong Province, Jinan, 250012 China; 2 UNC Project-China, Institute for Global Health and Infectious Diseases, University of North Carolina at Chapel Hill, Chapel Hill, NC USA; 3 SESH Global, Guangzhou, China; 4 Department of Epidemiology and Biostatistics, University of California, San Francisco, CA USA; 5 Department of Economics, Duke University, Durham, NC USA; 6 Shandong Provincial Center for Disease Control and Prevention, Jinan, China; 7 Guangdong Provincial Center for Skin Diseases and STI Control, Guangzhou, China

**Keywords:** HIV, Community engagement, Peer norm, Self-efficacy, Men who have sex with men, Path model

## Abstract

**Background:**

Community engagement strategies are often integrated in public health interventions designed to promote condom use among men who have sex with men (MSM), a key population for HIV prevention. However, the ways in which condom use peer norms and self-efficacy play a role in the association between community engagement and condom use is unclear. This study examines the potential mediating roles of peer norms and self-efficacy in this association.

**Methods:**

A nationwide cross-sectional online survey was conducted among Chinese MSM in 2015. Recruitment criteria included being born biologically male, being older than 16 years, having had anal sex with a man at least once during their lifetime, and having had condomless anal or vaginal sex in the past three months. Mplus 6.11 was used to conduct confirmatory factor analysis and path modeling analysis to examine the structural relationships between HIV/sexual health community engagement (e.g., joining social media and community events related to HIV and sexual health services), condom use peer norms, condom use self-efficacy, and frequency of condom use.

**Results:**

The study found that HIV/sexual health community engagement, condom use peer norms, condom use self-efficacy, and frequency of condom use were mutually correlated. A good data model was achieved with fit index: CFI = 0.988, TLI = 0.987, RMSEA = 0.032, 90% CI (0.028, 0.036). HIV/sexual health community engagement was associated with frequency of condom use, which was directly mediated by condom use peer norms and indirectly through self-efficacy.

**Conclusion:**

The study suggests that condom use peer norms and self-efficacy may be mediators in the pathway between community engagement and condom use, and suggests the importance of peer-based interventions to improve condom use.

**Electronic supplementary material:**

The online version of this article (doi:10.1186/s12889-017-4662-4) contains supplementary material, which is available to authorized users.

## Background

Meta-analyses of randomized controlled trials (RCTs) show that behavioral interventions have not been successful in sustainably changing condom use [1, 2]. Most of these interventions focused on individual-level behavior change and did not integrate community engagement. Community engagement is defined as a process of inclusive participation of community members in order to address issues that affect the well-being of their community [3]. This approach encourages participants to transform from being passive recipients of information to active problem-solvers [4]. Activists, volunteers, and peers are encouraged to organize events that are focused on education, prevention, and care, thus strengthening support networks among members of key populations, addressing social challenges of stigmatization and discrimination, and increasing community members’ perceptions of opportunities to participate in the promoted events [5, 6].

Community engagement is important for HIV/sexual health programs for three reasons: (1) it provides psychosocial benefits for participants, including increased senses of belonging [7] and empowerment [8], and decreased perceptions of stigma and isolation [9]; (2) it provides opportunities for capacity building [10]; and (3) it aids in the development of long-term community projects and networks, which remain in place after interventions end [11]. HIV/sexual health community engagement has been linked to increased condom use [12], increased HIV testing [13], improved linkage to care [14], greater access to treatment [15], and improved retention in care [16].

Studies have found a significant association between community engagement and individual-level condom use among MSM [17–20]. However, there is a relatively limited understanding of the factors that mediate this association, which may hinder efforts by health care professionals to develop effective community engagement campaigns that promote condom use. Studies have indicated that both peer norms and self-efficacy are important constructs in predicting human social behaviors and may serve as mediators between community engagement and condom use [6, 21–23]. Studies of MSM from Europe and the United States report positive intervention results in the form of safer sex practices, following efforts to strengthen mediators like norms and self-efficacy that facilitate behavioral change [24, 25].

Condom use peer norms are the expected patterns of behavior and attitudes toward condom use among one’s peers. Condom use self-efficacy is one’s confidence in one’s own ability to adhere to condom use guidelines in challenging situations [6, 23]. Peer norms are an important construct in the theory of reasoned actions and the theory of planned behavior, and self-efficacy is a core construct in social cognitive theory (SCT) [21, 26, 27]. Both peer norms and self-efficacy are strong predictors of condom use [28]. However, the theoretical constructs of peer norms and self-efficacy that apply in one context may not necessarily be applicable in another, given variation in factors like gender, ethnicity, and culture/subculture [29, 30].

In order to better understand how community engagement can contribute to the HIV response, it is important to investigate the ways in which community engagement influences peer norms and self-efficacy and promotes safe sexual behavior [17, 31, 32]. The purpose of this study is to answer the following questions, for a sample of Chinese MSM: does HIV/sexual health community engagement predict condom use or does it predict behavior via peer norms and self-efficacy? Second, are there indirect effects of community engagement on self-efficacy via peer norms? Third, are there direct effects of peer norms on condom use or indirect effects via self-efficacy?

## Methods

### Study population and procedure

A nationwide cross-sectional online survey of MSM was conducted in China from November 2 to 7 2015. In order to recruit participants from across the country, survey recruitment was done through popular online social networking platforms: Danlan.org, the largest gay web portal in China, and its associated gay mobile dating app; Weibo, a microblogging platform; and WeChat, a messaging app. Participants entered the survey by clicking on a banner ad, which directed them to a survey hosted on Qualtrics (Provo, Utah). The survey was anonymous and voluntary. Inclusion criteria included: being born biologically male, having had anal sex with a man at least once during their lifetime, having had condomless anal or vaginal sex in the past three months, and being at least 16 years of age. Informed consent was obtained from all eligible participants before they began the survey. Ethics approval was granted by institutional review boards at the Guangdong Provincial Centre for Skin Diseases and STI Control, China, the University of North Carolina at Chapel Hill, and the University of California, San Francisco.

### Measurements

The survey instrument was adapted from a previous online survey administered to Chinese MSM that included questions on sexual history, HIV/STI testing, and risk behaviors. The survey instrument was designed iteratively, with preliminary input from local stakeholders, sociologists, and physicians, as well as 60 MSM [33]. Survey questions were further modified based on the results of a comprehensive literature search, input from researchers who designed similar previous surveys, and two rounds of field-testing. A revised survey instrument was piloted with 150 MSM before the final survey was launched.

### HIV/sexual health community engagement

Community engagement was measured using an eight-item scale, adapted from community engagement literature [34–36] and piloted on 150 Chinese MSM. The scale items included: traditional engagement, which includes participation in volunteer activities that help others access HIV and sexual health services; and technology-based engagement, which includes watching videos and participating in discussions on social media about sexual health, HIV/STI prevention and care, and condom use (See Additional file 1: Table S1 for the detailed). Latent variable modeling was used to test the scale’s reliability and the corresponding alpha value was 0.720 (95% CI 0.692-0.749) [37]. The mean was calculated for the eight items, each of which had binary answers (Yes = 1, No = 0), with a possible high score of 1 and a possible low score of 0. Higher mean scores indicated higher self-reported levels of community engagement.

### Condom use peer norms

Peer norms for condom use were measured using a six-item scale [38]. Questions included participants’ perceptions of their friends’ attitudes towards condom use and safe sex. For example, participants were asked to evaluate the following statement: “If I had sex and told my friends that I did not use a condom, they would be angry or disappointed.” Answers were given in a five-point Likert format: strongly agree (5), agree (4), neutral (3), disagree (2), strongly disagree (1) (See Additional file 1: Table S2 for the detailed). In the present study, the Cronbach’s alpha value was 0.770. The mean was calculated with a possible high score of 5 and a possible low score of 1. Higher mean scores indicated higher self-reported strength of condom use peer norms.

### Condom use self-efficacy

Condom use self-efficacy was measured using a seven-item scale [39]. Participants were asked how comfortable they felt about negotiating and using a condom with sex partners. For example, participants were asked to evaluate the following statement: “I feel confident that I could refuse to have sex with a partner who did not want me to use a condom.” Answers were given in a five-point Likert format: Strongly agree (5), agree (4), neutral (3), disagree (2), strongly disagree (1) (See Additional file 1: Table S3 for the detailed). In the present study, the Cronbach’s alpha value was 0.823. The mean was calculated with a possible high score of 5 and a possible low score of 1. Higher mean scores indicated higher self-reported strength of condom use self-efficacy.

### Condom use

Frequency of condom use with four types of sex partners (primary male partner, casual male partner, primary female partner, and casual female partner) was evaluated using four survey items [40]. For example, participants were asked to evaluate the following statement: “In the last three months, when you had sex with a male casual partner, how frequently did you or your partner use condoms?” Answers were given in a four-point Likert format: Always used (4), mostly used (3), sometimes used (2), never used (1). In the present study, frequency of condom use was treated as an observed variable, instead of a latent variable [41]. The mean was calculated with a possible high score of 4 and a possible low score of 1. Higher mean scores indicated higher self-reported frequency of condom use.

### Statistical analyses

First, SPSS 19 was used to conduct a descriptive analysis of participants’ sociodemographic characteristics. Spearmen correlation tests were used to test the associations among the four variables (i.e., HIV/sexual health community engagement, condom use peer norms, condom use self-efficacy, and frequency of condom use). Second, Mplus 6.11 was used to conduct confirmatory factor analysis (CFA) to assess construct validity of the items and assess the goodness of fit of the measurement model. Third, we applied structural equation modeling (SEM) to examine the pathways of our hypothesized model. All items assumed to reflect latent factors (i.e., HIV/sexual health community engagement, condom use peer norm, and condom use self-efficacy) were defined as categorical variables. We used the robust weighted least squares (WLSMV) estimator, available in Mplus [42]. Fourth, we ran an initial model, which regressed condom use on community engagement, social norm, self-efficacy, and marital status (a sociodemographic variable related to condom use in correlation analysis). Results indicated that the coefficients on “condom use on community engagement” and “condom use on peer norm” were not significant at *p* < 0.1. For subsequent analyses, we removed these non-significant regression paths. Fifth, modification indices (MI > 25) were examined to identify missing paths and seven covariate paths among indicators were added step by step. We then reached the final good data model. The overall model fit was examined by using the comparative fit index (CFI) and the root mean square error of approximation (RMSEA). For the CFI, values greater than 0.95 indicate a good model fit, and for RMSEA, a value below 0.06 indicates good fit [43]. The indirect effects were calculated using the Delta method in Mplus [42].

## Results

In total, 7892 people clicked the banner link to the survey, and 7551 (96%) began the survey. Of the 1597 participants who met inclusion criteria and provided informed consent, 1189 participants completed the survey. However, 147 participants did not answer any questions about frequency of condom use. A total of 1042 participants were included in the current data analysis.

### Sample characteristics

Among the 1042 participants, the mean age was 25.3 ± 6.77; 62.4% were ≤25 years of age; 13.3% were currently married to a woman or engaged; 35.0% were students; 67.6% had a college diploma or higher level of education; 81.1% had a monthly income less than 806.5 USD (5000 RMB); 70.3% identified as gay while 25.9% identified as bisexual (Table 1). A descriptive cross-table for sociodemographic variables, community engagement, peer norms, self-efficacy, and condom use is also provided (see Additional file 1: Table S4).

**Table 1:** Sociodemographic characteristics of online high-risk MSM in China, 2015 (*n* = 1042)

Characteristics	Frequency (*N* = 1042)	Percentage (%)
Age^a^		
≤ 25	648	62.4
26–35	306	29.5
36–45	67	6.4
≥ 46	18	1.7
Marital status		
Not married	853	81.9
Engaged or Married	139	13.3
Separated or Divorced	48	4.6
Widowed	2	0.2
Student status		
Yes	365	35.0
No	677	65.0
Education level		
High school or below	338	32.4
College diploma	264	25.3
Undergraduate	390	37.4
Postgraduate (Master/PhD)	50	4.8
Individual monthly income		
< 1500 RMB (241.9 USD)	276	26.5
1500–3000 RMB (242–483.9 USD)	269	25.8
3001–5000 RMB (484–806.5 USD)	300	28.8
5001–8000 RMB (806.6-1290 USD)	100	12.5
> 8000 RMB (1290 USD)	67	6.4
Sexual identity		
Gay	733	70.3
Bisexual	270	25.9
Straight/Heterosexual	1	0.1
Unsure/Other	38	3.6

^a^Age: mean = 25.3, SD = ± 6.767(with three missing values)

### Bivariate correlations between community engagement, peer norms, self-efficacy and condom use

The mean score for community engagement was 0.35 (SD = 0.25). The mean score for condom use peer norms was 3.75 (SD = 0.71). The mean score for condom use self-efficacy use was 3.98 (SD = 0.68). The mean score for condom use was 2.37 (SD = 1.00) (Table 2). Results in Table 3 indicate that the predictor variable of community engagement was significantly correlated with both mediators, condom use peer norms (*r*_*s*_ = 0.152, *p* < 0.001) and condom use self-efficacy (*r*_*s*_ = 0.140, *p* < 0.001), and the outcome variable of condom use (*r*_*s*_ = 0.085, *p* < 0.01). Furthermore, the two mediators were also significantly correlated with the outcome variable (*r*_*s*_ = 0.148, *p* < 0.001; *r*_*s*_ = 0.254, *p* < 0.001), supporting the proposed mediation model.

**Table 2:** Descriptive statistics of four variables

	Mean(SD)	Min	Q25	Median	Q75	Max	N
Engagement	0.35 (0.25)	0.00	0.13	0.25	0.5	1.00	1042
Peer norms	3.75 (0.71)	1.00	3.33	3.67	4.33	5.00	1042
Self-efficacy	3.98 (0.68)	1.43	3.57	4.00	4.57	5.00	1042
Condom use	2.37 (1.00)	1.00	1.50	2.00	3.00	4.00	1042

**Table 3:** Correlations between variables of community engagement, peer norms, self-efficacy and condom use among online high-risk MSM in China, 2015 (*n* = 1042)

Variable	2	3	4
1.community engagement	0.152***	0.140***	0.085**
2.peer norms		0.516***	0.148***
3.self-efficacy			0.254***
4.condom use			

*** *p* < 0.001, ** *p* < 0.01

### Measurement model of community engagement, peer norms and self-efficacy

Confirmatory factor analysis showed that all items loaded significantly on their corresponding factors (all loadings *P* < 0.01) (Table 4). A test of the measurement model resulted in the following fit index: CFI = 0.960, TLI = 0.954, RMSEA = 0.064, 90% CI (0.060, 0.068), indicating good fit. Standardized factor loading of the modified measurement model ranged from 0.229 to 0.935. All were statistically significant at *p* < 0.001.

**Table 4:** Unstandardized and standardized loading for measurement model of community engagement, peer norms and self-efficacy among online high-risk MSM in China (*n* = 1042)

Parameter estimate	Unstandardized loading(*SE*)	Standardized loading
Engagement → I1	1.000	0.809
Engagement → I2	0.902 (0.052)***	0.745
Engagement → I3	0.700 (0.045)***	0.566
Engagement → I4	1.088 (0.047)***	0.880
Engagement → I5	1.088 (0.052)***	0.880
Engagement → I6	0.667 (0.051)***	0.539
Engagement → I7	0.697 (0.051)***	0.564
Engagement → I8	0.580 (0.053)***	0.469
Peer norms → F3	1.000	0.229
Peer norms → F4	2.507 (0.336)***	0.574
Peer norms → F5	2.650 (0.356)***	0.606
Peer norms → F6	3.740 (0.494)***	0.856
Peer norms → F7	3.827 (0.501)***	0.875
Peer norms → F8	4.086 (0.538)***	0.935
Self-efficacy → F9	1.000	0.727
Self-efficacy → F10	0.932 (0.033)***	0.678
Self-efficacy → F11	0.963 (0.033)***	0.700
Self-efficacy → F12	1.048 (0.031)***	0.761
Self-efficacy → F13	0.993 (0.029)***	0.721
Self-efficacy → F14	0.958 (0.030)***	0.696
Self-efficacy → F15	0.954 (0.031)***	0.694

As suggested by the modification indices, adjustment of original CFA model is not needed

Standard errors are in the parenthesis

*** *p* < 0.001, Fit index: CFI = 0.960, TLI = 0.954, RMSEA = 0.064, 90% CI (0.060, 0.068)

### Structured Path Model of Community Engagement, Peer Norms, Self-Efficacy and Condom Use

**Fig. 1:**
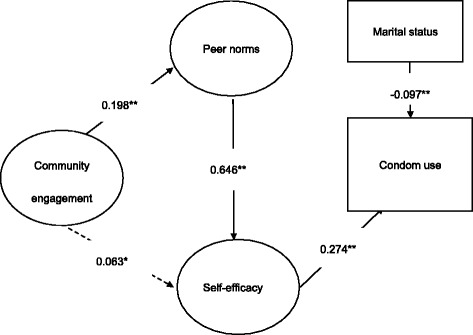
Structured path model of community engagement, peer norms, self-efficacy and condom use among online high-risk MSM in China, 2015 (*n* = 1042). Path model of community engagement, peer norm, self-efficacy, and condom use ** *p* < 0.001, * *p* < 0.1. Note: Fit index: CFI = 0.988, TLI = 0.987, RMSEA = 0.032, 90% CI (0.028, 0.036). Only significant routes were included in the figure. All path coefficients and factor loadings shown were standardized

Results in Fig. 1 indicate a good data model with fit index: CFI = 0.988, TLI = 0.987, RMSEA = 0.032, 90% CI (0.028, 0.036). Community engagement was associated with condom use peer norms (*b* = 0.198, *p* < .001) and marginally associated with self-efficacy (*b* = 0.063, *p* < .1). The two mediators were significantly associated with each other (*b* = 0.646, *p* < .001). Only self-efficacy was significantly associated with the outcome variable (*b* = 0.274, *p* < .001). The model explained 8.5% of the variance in condom use. The estimated mediation effect between community engagement and condom use via peer norms and self-efficacy was 0.052 (*p* < 0.001; Table 5). The mediation effects via specific paths are shown in Table 5.

**Table 5:** Indirect effects of community engagement on condom use via peer norms and self-efficacy among online high-risk MSM in China, 2015 (*n* = 1042)

Pathways to condom use	Unstandardized *β*	*SE*	*P* value	Standardized *β*
Engagement - self-efficacy - condom use	0.040	0.024	0.099	0.017
Engagement-peer norms - self-efficacy –condom use	0.081	0.023	0.000	0.035
Total indirect effect of engagement	0.121	0.033	0.000	0.052
Peer norms - self-efficacy - condom use	0.757	0.142	0.000	0.177

With WLSMV, Mplus doesn’t provide standard errors and *p*-values for standardized estimates when the model contains covariates

## Discussion

This study tested the proposed mediating roles of condom use peer norms and self-efficacy in the association between HIV/sexual health community engagement and frequency of condom use. The hypothesized structural path model was tested using data from a nationwide online survey with a sample of 1042 sexually active MSM in China. The proposed mediation relationship was supported by correlation analysis and confirmed by structural path modeling analysis, which support the need for additional longitudinal studies to assess causality in the relationship. The study findings add to the existing literature by demonstrating the potential indirect effect of community engagement on self-efficacy via peer norms, and the potential indirect effect of peer norms on condom use via self-efficacy. This study provides insight about the ways in which future public health interventions geared towards condom use ought to be designed.

The results suggest that HIV/sexual health community engagement may not directly impact condom use self-efficacy among sexually active MSM in China. Instead, community engagement may indirectly impact condom use self-efficacy via condom use peer norms. Scholars have argued that the Chinese are socialized to adhere to social norms and cultural rules, with peer norms influencing individuals’ beliefs, attitudes, and behaviors [44, 45]. Among sexually active Chinese MSM, ethnographies have found that peer norms in some gay settings influenced subjects’ beliefs and self-efficacy in regard to condom use [46, 47]. When MSM participate in HIV/sexual health community events and, subsequently, develop positive perceptions of their friends’ attitudes towards condom use and safe sex, they may improve their self-efficacy for condom use. Stronger perceptions of peer norms may increase one’s self-efficacy in regard to condom use [48, 49]. This is a potential way in which community engagement may influence self-efficacy by altering perceptions of peer norms regarding condom use.

The results of this study also suggest that within the association between community engagement and condom use, peer norms may have an indirect effect on condom use via self-efficacy. This finding extends our knowledge about these relationships from previous studies, in which the existence of peer norms and self-efficacy were reported only as predictors of condom use [28, 50]. Studies have identified self-efficacy alone as a mediator of the intervention effect for condom use [24, 49, 51]. Moreover, a study showed that while peer norms and self-efficacy were both significantly associated with condom use in bivariate analysis, only self-efficacy remained significant in multivariate analysis [48]. Our path analysis suggests that peer norms are not sufficient to influence condom use directly. While self-efficacy for condom use may be a core mediator, peer norms act through self-efficacy in order to impact condom use. The central role of self-efficacy in a mediating relationship has been confirmed, which is consistent with other empirical mediating analyses on self-efficacy, [49, 52] as well as theoretical arguments [53, 54].

The present study has implications for the design of public health interventions. First, our results suggest that HIV/sexual health community engagement can enhance condom use peer norms and self-efficacy, thus impacting condom use behavior. These findings encourage integrating community engagement approaches into HIV prevention programs. Previous studies have examined only traditional approaches to community engagement and excluded online engagement through social media platforms [12, 17, 20]. Our study suggests including online and social media activities related to HIV/sexual health and condom use alongside traditional community engagement approaches. This is an active response to the rapid development of online communities and social media interactions among MSM in China. Moreover, the integration of both traditional community events and online social media activities may offer broader opportunities to address stigma and discrimination against MSM. Other studies have also encouraged these combined approaches to interventions [55, 56].

Second, our study suggests that community engagement has an indirect effect on condom use by impacting peer norms and self-efficacy for condom use. When community engagement is utilized to promote condom use, it is important to include concepts of peer norms and self-efficacy for condom use into the design and programming of the intervention campaign. Our study also encourages the integration of peer intervention [25, 57] programs within community engagement campaigns in order to strengthen condom use peer norms and self-efficacy.

Our study has several limitations. First, this analysis relied on cross-sectional data, limiting our ability to assess causality. Second, the data were obtained from an online survey with participants who tended to be relatively young and well educated. Moreover, we only recruited high-risk MSM, which limited our ability to assess the MSM population in China, but allowed the analysis to focus on HIV prevention for the key population. Finally, the frequency of condom use was measured using self-reported data, so social desirability bias may be a concern.

## Conclusions

In sum, our study contributes to the literature by supporting and presenting the potential mediating roles of condom use peer norms and self-efficacy between HIV/sexual health community engagement and frequency of condom use. This study identifies the indirect effect of community engagement on self-efficacy via peer norms and the indirect effect of peer norms on condom use via self-efficacy. In terms of intervention design, the results indicate that effective community engagement campaigns should incorporate both online social media activities and traditional community events in order to address stigma and discrimination against MSM. The study encourages adopting peer interventions to promote condom use.

## Additional file

Additional file 1: Table S1. Eight items of HIV/ sexual health community engagement among high-risk MSM in China, 2015 (*n* = 1042). Table S2. Six items of condom use peer norm among high-risk MSM in China, 2015 (*n* = 1042). Table S3. Seven items of condom use self-efficacy among high-risk MSM in China, 2015 (*n* = 1042). Table S4 Descriptive cross-table for sociodemographic variables, community engagement, peer norms, self-efficacy, and condom use (DOCX 20 kb)

## Abbreviations

CFA: Confirmatory factor analysis
CFI: Comparative fit index
CI: Confidence interval
HIV: Human immunodeficiency virus
MI: Modification indices
MSM: Men who have sex with men
RCT: Randomized controlled trial
RMB: Ren min bi
RMSEA: Root mean square error of approximation
SD: Standardized deviation
SEM: Structural equation modeling
SPSS: Software package for statistical analysis
STI: Sexually transmitted infections
TLI: Tucker Lewis index
USD: United states dollar
WLSMV: Robust weighted least squares
