# Supplementary material for: Condom use peer norms and self-efficacy as mediators between community engagement and condom use among Chinese men who have sex with men
Source: BMC Public Health. 2017 Aug 7;17:641. doi: 10.1186/s12889-017-4662-4 (PMC5545844; doi:10.1186/s12889-017-4662-4)
Supplement: Additional file 1: Table S1. — Eight items of HIV/ sexual health community engagement among high-risk MSM in China, 2015 (n = 1042). Table S2. Six items of condom use peer norm among high-risk MSM in China, 2015 (n = 1042). Table S3. Seven items of condom use self-efficacy among high-risk MSM in China, 2015 (n = 1042). Table S4 Descriptive cross-table for sociodemographic variables, community engagement, peer norms, self-efficacy, and condom use (DOCX 20 kb) [file 12889_2017_4662_MOESM1_ESM.docx]

**Additional file**

**Table 1. Eight items of HIV/ sexual health community engagement among high-risk MSM in China, 2015 (n = 1042)**

| I1 | In the last three weeks, have you viewed any videos promoting condom use among MSM? | 1=Yes, 0=No |
| --- | --- | --- |
| I2 | In the last three weeks, have you viewed any videos promoting HIV testing among MSM? | 1=Yes, 0=No |
| I3 | Are you aware of any ongoing community events promoting sexual health among MSM? | 1=Yes, 0=No |
| I4 | Have you ever helped organize a testing and/or awareness campaign (e.g. HIV, condom use, etc.) that promoted sexual health among MSM? | 1=Yes, 0=No |
| I5 | Have you ever volunteered at a health clinic or other location that provided sexual health services among MSM? | 1=Yes, 0=No |
| I6 | Have you ever encouraged someone else to get tested for HIV and/or another sexually transmitted disease? | 1=Yes, 0=No |
| I7 | Have you ever accompanied a friend or partner to a testing facility to get tested for HIV and/or another sexually transmitted disease? | 1=Yes, 0=No |
| I8 | Have you ever participated in online forums or discussions on social media (ie. Weixin, Weibo, Twitter, or other on-line communities) about about sexual health, condom use, or HIV/STD testing or related services? | 1=Yes, 0=No |

**Table 2. Six items of condom use peer norm among high-risk MSM in China, 2015 (n = 1042)**

| F3 | If I had sex and told my friends that I did not use a condom, they would be angry or disappointed. | 5=Strongly agree, 4=Agree, 3=Neutral, 2=Disagree, 1=Strongly disagree |
| --- | --- | --- |
| F4 | My friends talk a lot about “safer" sex. | 5=Strongly agree, 4=Agree, 3=Neutral, 2=Disagree, 1=Strongly disagree |
| F5 | My friends and I encourage each other before dates to practice "safer" sex. | 5=Strongly agree, 4=Agree, 3=Neutral, 2=Disagree, 1=Strongly disagree |
| F6 | If I thought that one of my friends had sex on a date, I would ask them if they used a condom. | 5=Strongly agree, 4=Agree, 3=Neutral, 2=Disagree, 1=Strongly disagree |
| F7 | If a friend knew that I might have sex on a date, he/she would ask me if I was carrying a condom. | 5=Strongly agree, 4=Agree, 3=Neutral, 2=Disagree, 1=Strongly disagree |
| F8 | When I think that one of my friends might have sex on a date, I would ask him/her if he/she was carrying a condom. | 5=Strongly agree, 4=Agree, 3=Neutral, 2=Disagree, 1=Strongly disagree |

**Table 3. Seven items of condom use self-efficacy among high-risk MSM in China, 2015 (n = 1042)**

| F9 | If I might have sex on a date and I do not have a condom, I would make an effort to go out of my way and get one. | 5=Strongly agree, 4=Agree, 3=Neutral, 2=Disagree, 1=Strongly disagree |
| --- | --- | --- |
| F10 | I would feel comfortable discussing condom use with a potential partner before we engaged in sex. | 5=Strongly agree, 4=Agree, 3=Neutral, 2=Disagree, 1=Strongly disagree |
| F11 | I would feel comfortable letting a primary partner know that I want to have sex with a condom. | 5=Strongly agree, 4=Agree, 3=Neutral, 2=Disagree, 1=Strongly disagree |
| F12 | I would feel comfortable letting a casual partner know that I want to have sex with a condom | 5=Strongly agree, 4=Agree, 3=Neutral, 2=Disagree, 1=Strongly disagree |
| F13 | I feel confident that I could refuse to have sex with a partner who did not want you to use a condom | 5=Strongly agree, 4=Agree, 3=Neutral, 2=Disagree, 1=Strongly disagree |
| F14 | I feel confident in my ability to incorporate putting a condom on myself or my partner into foreplay. | 5=Strongly agree, 4=Agree, 3=Neutral, 2=Disagree, 1=Strongly disagree |
| F15 | I feel confident that I could use a condom with a partner without "breaking the mood." | 5=Strongly agree, 4=Agree, 3=Neutral, 2=Disagree, 1=Strongly disagree |

**Table 4 Descriptive cross-table for sociodemographic variables, community engagement, peer norms, self-efficacy, and condom use**

|  | Community engagement | Peer norms | Self-efficacy | Condom use |
| --- | --- | --- | --- | --- |
| Age |  |  |  |  |
| ≤25 | 0.35 | 3.67 | 3.91 | 2.42 |
| 26-35 | 0.36 | 3.90 | 4.12 | 2.32 |
| 36-45 | 0.33 | 3.95 | 4.22 | 2.18 |
| ≥46 | 0.34 | 3.42 | 3.53 | 2.02 |
| Marital status |  |  |  |  |
| Not married | 0.35 | 3.74 | 3.96 | 2.43 |
| Engaged or Married | 0.37 | 3.81 | 4.10 | 2.09 |
| Separated or Divorced | 0.35 | 3.78 | 3.97 | 2.15 |
| Widowed | 0.50 | 3.17 | 3.93 | 3.50 |
| Students status |  |  |  |  |
| Yes | 0.37 | 3.72 | 3.90 | 2.44 |
| No | 0.34 | 3.76 | 4.03 | 2.34 |
| Educational level |  |  |  |  |
| High school or below | 0.34 | 3.71 | 3.92 | 2.28 |
| College diploma | 0.34 | 3.76 | 4.00 | 2.49 |
| Undergraduate | 0.36 | 3.76 | 4.01 | 2.38 |
| Postgraduate (Master/PhD) | 0.41 | 3.83 | 4.04 | 2.30 |
| Individual monthly income |  |  |  |  |
| <1500 RMB (241.9 USD) | 0.35 | 3.66 | 3.87 | 2.42 |
| 1500-3000 RMB (242-483.9 USD) | 0.34 | 3.75 | 3.94 | 2.34 |
| 3001-5000 RMB (484-806.5 USD) | 0.34 | 3.82 | 4.09 | 2.43 |
| 5001-8000 RMB (806.6-1290 USD) | 0.36 | 3.75 | 4.03 | 2.37 |
| >8000 RMB (1290 USD) | 0.43 | 3.77 | 4.05 | 2.06 |
| Sexual identity |  |  |  |  |
| Gay | 0.35 | 3.78 | 4.00 | 2.36 |
| Bisexual | 0.34 | 3.67 | 3.95 | 2.42 |
| Straight/Heterosexual* | 0.13 | 4.33 | 3.57 | 1.00 |
| Unsure/Other | 0.35 | 3.72 | 3.95 | 2.33 |

* Only 1 person thought he was straight/heterosexual.
